# The effects of cyber-ostracism on college students' aggressive behavior: a moderated mediation model

**DOI:** 10.3389/fpsyt.2024.1393876

**Published:** 2024-04-18

**Authors:** Jingwen Xing, Fengyi Kuo

**Affiliations:** ^1^ School of Primary Education, Shanghai Normal University Tianhua College, Shanghai, China; ^2^ School of Rehabilitation, Jiangsu Vocational College of Medicine, Yancheng, China

**Keywords:** cyber-ostracism, self-integrity, aggressive behavior, basic psychological needs, a moderated mediation model

## Abstract

**Purpose:**

This study used questionnaire survey to explore the influence of cyber-ostracism on the aggressive behavior of college students. Specifically, this study explored the mediation role of the basic psychological needs satisfaction, and explored the moderating role of self-integrity.

**Method:**

An online questionnaire was designed through a questionnaire website, which was linked and transferred to college students nationwide. 377 valid questionnaires were obtained after excluding invalid questionnaires.

**Results:**

Cyber-ostracism had a significant positive predictive effect on the basic psychological needs satisfaction; Basic psychological needs satisfaction play a mediation role between cyber-ostracism and aggression. Self-integrity moderates the association between basic psychological needs and aggression.

## 1 Introduction

As a symbol of a low level of social adaption, aggressive behaviors are usually antisocial and have a wide negative influence, which is a hot research topic in the field of mental health (1). According to a report from World Health Organization (WHO), teenagers of 15-29 were responsible for about 37% of global violent crimes (2). College students are in a transition period, when they are changing from a teenager to an adult, leaving school while stepping into society, and their attacking behaviors are more anonymous and complicated. So, the ignorance of college student’s attacking behaviors may lead to more severe violent crimes and social problems. Hence the research focuses on the mechanism and protecting factors of college student’s aggressive behavior.

According to Erikson’s theory of personality development, individual’s key developing mission during early adulthood is solving the conflict between intimacy and loneliness (3). And college students are in such an important developing period, they need to develop good relationships with others. However, it’s unavoidable that individuals may face obstacles when developing social relationship with others, and ostracism is a common negative event (4). Ostracism refers to the phenomenon that an individual is refused or neglected by other individuals or groups, which can have a passive effect on individual’s physical and mental health and behaviors, like physiological and mental pain, anxiety, depression and aggressive behavior (5). In the past, people mainly develop social relations through face to face interactions, fulfilling their needs of belonging. However, the fast development of the Internet let people communicate with others online more. Hitherto, the Internet has become an indispensable social media, and ostracism also happens in cyberspace, which refers to cyber-ostracism (5, 6). According to 51^st^ Statistical Report on China's Internet Development, there are citizens using the Internet, among which college students accounts for the main population (7). Internet has become an important tunnel for college students to socialize and gain new knowledge, but the anonymity, uncertainty of location and time and lack of social clue of the Internet lead to the high incidence of cyber-ostracism among college students, which needs our attention.

### 1.1 Cyber-ostracism and aggressive behavior

Ostracism refers to the negative feeling that arises when individuals don’t receive reactions from others during a certain period of time, and they feel being ignored or rejected (4). Cyber-ostracism happens when individual don’t receive communication and affirmation that he or she desires during an acceptable period of time in face-to-face electronic media interaction. The main media of cyber-ostracism include e-mail, social website, online chatting room, instant messaging tools and so on (4, 5). For example, individuals don’t receive replies from others after talking in online chatting room, or they receive no comments or likes after posting new contents in social websites (6).

Former research results showed many similarities between real-life ostracism and cyber-ostracism. Similar to social exclusion, cyber-ostracism also brought individual painful experiences, making regions in brain relevant to physical pain more active (8), leading to threats against basic psychological needs and negative emotions (9–11). However, compared with offline interactions, interactions in cyberspace are anonymous, which can depersonalize individuals easily, and subsequently decrease their control of behaviors, making them more prone to perform antisocial behaviors, like harming group cooperation and aggressive behaviors (10, 12). Besides, individuals experiencing cyber-ostracism didn’t need to worry about social situations in real life (13), which made them prone to deride from moral constraints and behave antisocially.

According to self-control failing theory, individual’s self-control resources are limited, and ostracism may decrease individual’s self-control resources, leading to antisocial behaviors (14, 15). In addition, the premise of normal social communication in society is to have the ability of self-control, which is the psychological resources for individuals to control the desire that does not meet the social expectations (15). Network social exclusion will also damage the self-control ability of individuals, leading to the occurrence of habituation and impulsive behaviors. At the same time, it is also easy to continue to be rejected by others in the network society due to the weakening of self-control ability, so such a vicious circle leads to an increase in the probability of aggressive behavior.

Ostracism may lead to aggressive behavior through different psychological variables, like hostile cognitions, malicious envy, feelings of rule negligence and relative deprivation (It can be found that existing researches paid more attention to those negative mental mechanisms, while positive factors were less highlighted. Hence, the present study explored the possible mediating role of positive factors in the relationship, and basic psychological needs was chosen due to its importance in predicting individual behavioral responses. Besides, reducing the negative effects of psychological threat on individuals (16), self-integrity was deemed as an important moderator in the relationship.

### 1.2 The mediating role of basic psychological needs satisfaction

Self-determination theory points out that basic psychological needs satisfaction are necessary for individuals to achieve happiness and grow, and are also important predictors of individual behavioral responses (17). The demand threat time model states that individuals experience pain during the reflex phase and more negative emotions after social rejection and basic psychological needs, and then evaluate and determine the corresponding behavioral response during the reflection phase (5). Meeting basic psychological needs promotes the psychological growth and happiness, while blocking basic psychological needs increases the risk of psychological pathology and defensive reactions (17, 18). For example, the basic psychological needs satisfaction can increase self-esteem (19), self-realization (20), etc., So that a person can grow up comprehensively and healthily; Basic psychological needs satisfaction are predictors of learning motivation, dropout, anxiety and antisocial behavior (21–24). Besides basic psychological needs satisfaction plays a mediating role between cyber-ostracism and psychological well-being (25). And certain elements in video games that hinder satisfying the player’s basic psychological needs, this leads to aggression (26).

Individuals experience pain during the reflex phrase after social rejection, and they also feel more negative emotions and unfulfilled basic psychological needs, leading them to evaluate and determine corresponding behaviors (5). Many studies revealed that cyber-ostracism threatened the fundamental needs hugely (27, 28). At the same time, empirical studies showed that basic psychological needs mostly appeared in the form of mediating variables. For instance, a study found that parenting style influenced teenager’s social adaptation indirectly through the mediating mechanism of basic psychological needs satisfaction (29). Certain elements in video games hindered the satisfaction of player’s basic psychological needs, which led to aggression (26). Wang et al. (25) found that satisfaction of basic psychological needs mediated the effects of online social exclusion on two types of well-being. Based on this, this study speculated that basic psychology needs to play a mediating role in the relationship between cyber-ostracism and aggressive behavior.

### 1.3 The moderating role of self-integrity

Steele (30) first put forward the concept of self-integrity (self-integrity). Self-integrity means that even if they still have deficiencies in some aspects, they are generally good and adapted to the society. The self-affirmation theory states that people usually pursue a general good self-evaluation, that is, the perception of self-integrity rather than a specific self-evaluation, so everyone has the motivation to maintain self-integrity (30, 31). In life, we often encounter situations threatening self-integrity. When faced with such threat situations, individuals can avoid damage to self-integrity by affirming their self-value unrelated to the threat field (30, 31). In today’s digital age, cyber-ostracism is a common situation that threatens self-integrity. After the cyber-ostracism, individuals will feel pain and doubt the value of their own existence, and feel the basic psychological needs are blocked (32). If the individual affirms his self-worth in other fields unrelated to cyber-ostracism to maintain his self-integrity, he can have a constructive thinking to face the cyber-ostracism and basic psychological needs satisfaction, and think about how he can improve himself in the future without any defensive reactions such as aggressive behavior (31). Compared with individuals with low self integrity, individuals with high self integrity are often able to deal with self-threatening situations in a more active and socially adaptive way, thus avoiding defensive responses. In addition, previous studies have found that self-integrity does not directly reduce the psychological threat of individuals, but can reduce the negative effects of psychological threat on individuals (16). Therefore, this study believes that self-integrity can buffer the influence of cyber-ostracism on aggressive behavior

### 1.4 The current study

**Figure 1:**
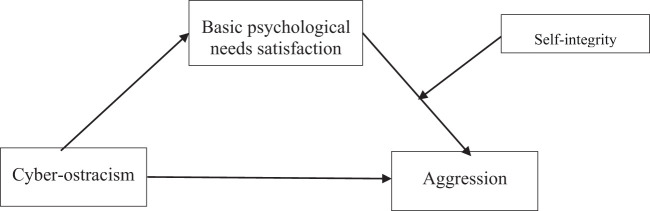
The hypothesized moderating mediation model.

In conclusion, combined with the self-affirmation theory and the self-determination theory, the present study constructed a moderated mediation model (see **Figure 1**) to examine the mechanisms underlying the relationship between cyber-ostracism and aggressive behavior among undergraduate students. Based on relevant empirical and theoretical evidence, the following hypotheses were pointed out:

H1: The experience of cyber-ostracism may significantly and positively predict the aggressive behavior of college students;

H2: The basic psychological needs satisfaction may play a mediating role in the influence of cyber-ostracism on college students’ aggressive behavior;

H3: Self-integrity may play a moderating role in the influence of cyber-ostracism on college students ‘aggression. Specifically, high self-integrity may buffer the influence of network social exclusion on college students’ aggressive tendency

## 2 Methods

### 2.1 Participants

A total of 377 college students were recruited randomly (through convenience sampling), who were asked to participate in an online questionnaire survey. Among them, 182 were boys, accounting for 48.3%, and 195 were girls, accounting for 51.7%. Age ranged from 18 to 27. All participants signed informed consent before participating in the study.

### 2.2 Measurements

#### 2.2.1 The cyber-ostracism questionnaire

The cyber-ostracism questionnaire compiled by Tong (33) was used to measure the cyber-ostracism in individuals’ daily life. The questionnaire consists of 14 items, with the example question “I chat with each other online, it is difficult to get an enthusiastic response”, using 5 points to score, and 1 representative never, 5 represents always. Higher scores on the questionnaire indicate a higher frequency of individuals experiencing online social exclusion in daily life. The Cronbach’s α of this questionnaire in this study was 0.92.

#### 2.2.2 The self-integrity scale

The self-integrity of individuals was measured using the Self-Integrity Scale (Self-Integrity Scale) (34). This scale was translated from the English version and also had good internal consistency in the Chinese subject population, Cronbach’s α =0.88 (35). There are 8 items in this scale, the example “I think I am basically a moral person”, 7 points, 1 represents very disagree, and 7 represents very agree. Higher scale scores indicate higher self-integrity of individuals. The Cronbach’s α of this scale in this study was 0.93.

#### 2.2.3 The simplified basic psychological needs satisfaction scale

The simplified basic psychological needs satisfaction scale (36). There are 9 items in the scale, with examples “I feel capable and capable”, “I feel controlled and stressed”, 7 points, 1 is very inconsistent, 7 is very consistent. The higher the score, the higher the satisfaction of basic psychological needs. The Cronbach’s α of this scale in this study was 0.92.

#### 2.2.4 The aggressive scale

Lu et al. (37) revised Chinese college version of aggressive scale, the Cronbach’s of the scale in college students was 0.89. A total of 22 items include hostility, impulse, irritability and physical aggression four dimensions, including hostility (8 items) examples such as “sometimes I feel others laugh at me”, impulsive (6 items) examples such as “when others disagree with me, I can’t help but argue with them”; irritability (3 items) cases such as “sometimes I get angry for no reason”, physical attack (5 items) examples such as “by provocation I may beat each other”. With 5 points, 1 is very inconsistent, and 5 is very consistent, and the higher the scale score, the more aggressive the individual is. The Cronbach’s α of this scale in this study was 0.93.

### 2.3 Data analysis

Data were collated and analyzed using the office software Excel and the data statistical analysis software SPSS25.0. Mediation analysis with moderation was performed using the process3.3 macro program on SPSS.

### 2.4 Common method deviation test

The Harman univariate method was used to test the possible common method bias problems in this study. The results showed that the first factor explained 31.1% of the variation, below the critical value of 40% (38). Therefore, it can be considered that this study does not have a serious problem of common methodological bias.

## 3 Results

### 3.1 Descriptive statistics and correlation analysis

Descriptive statistics and correlation analysis were performed for core variables, gender, age, and frequency of social networking sites (SNS) use. There was a significant negative correlation between cyber-ostracism and basic psychological needs satisfaction (*r*=−0.55, *p*<0.01), cyber-ostracism was significantly positively correlated with aggression (*r*=0.45, *p*<0.01); Basic psychological needs satisfaction was positively correlated with self-integrity (*r*=0.51, *p*<0.01), basic psychological needs satisfaction was negatively correlated with aggression (*r*=−0.48, *p*<0.01); Self-integrity was negatively correlated with aggression (*r*=−0.30, *p*<0.01), and the results are shown in **Table 1**.

**Table 1:** Descriptive statistics and correlations between variables.

Variables(*N*=377)	*M±SD*	1	2	3	4	5	6	7
1 Cyber-ostracism	2.64±0.95	1.00						
2 Basic psychological needs satisfaction	4.64±1.43	−0.55^**^	1.00					
3 Self-integrity	4.88±1.61	−0.09	0.51^**^	1.00				
4 Aggression	2.52±0.79	0.45^**^	−0.48^**^	−0.30^**^	1.00			
5 Gender		−0.06	0.13^*^	0.18^**^	−0.03	1.00		
6 Age	21.35±1.84	0.02	−0.01	0.01	0.01	0.02	1.00	
7 Frequency of SNS use	2.93±1.10	−0.03	0.18^**^	0.24^**^	−0.13^*^	0.17^**^	0.09	1.00

*p <.05. **p <.01. ***p <.001; Gender: 0= male, 1= female; Frequency of SNS use: 1 = 1 or less times per day, 4= 10 or more times per day.

### 3.2 The mediating effect of basic psychological needs satisfaction

To examine the mediating role of basic psychological needs satisfaction between cyber-ostracism and aggression. The process3.3 macro program in SPSS25.0 was used to conduct the mediation analysis, model 4 in process was selected, the sampling times of Bootstrap was set to 5000 times, and the confidence interval was set to 95%. Gender, age and frequency of SNS use as the control variables, and we standardized all the variables. The analysis was carried out according to the mediating effect test procedure, the results were shown in **Tables 2**, **3**.

**Table 2:** Testing the mediating effect of cyber-ostracism and aggression.

Model (*N*=377)		Fit index			Significance	
Outcome variable	Predictor variable	*R*	*R^2^*	*F*	*β*	*t*
Basic psychological needs satisfaction		0.58	0.33	46.77^***^		
	cyber-ostracism				−0.54	−12.82^***^
	Gender				0.14	1.611
	Age				−0.01	−0.28
	Frequency of SNS use				0.14	3.43^***^
Aggression		0.53	0.28	28.78^***^		
	Cyber-ostracism				0.27	5.06^***^
	Basic psychological needs satisfaction				−0.32	−5.92^***^
	Gender				0.07	0.82
	Age				0.01	0.10
	Frequency of SNS use				−0.07	−1.54

**Table 3:** Total, direct and indirect effects.

	Relative value	*SE*	Bootstrap LLCI	Bootstrap ULCI
Indirect effects	0.17	0.03	0.11	0.24
Direct effects	0.27	0.05	0.16	0.37
Total effects	0.44	0.05	0.35	0.53

**Figure 2:**
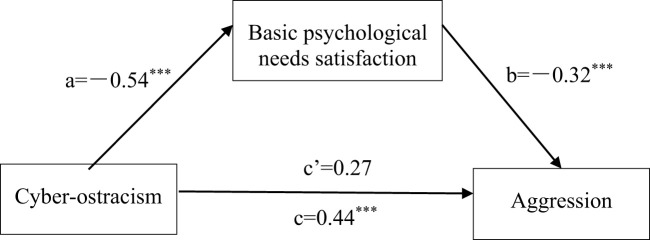
The mediating effect of cyber-ostracism and aggression. ***p <.001.

The results in **Table 2** shown that cyber-ostracism had a significant positive predictive effect on the basic psychological needs satisfaction (*β*=−0.54, *t=*−12.82, *p*<0.001); Satisfaction of basic psychological needs had a significant negative predictive effect on aggression (*β*=−0.32, *t*=−5.92, *p*<0.001); cyber-ostracism significantly positively predicted aggression (*β*=0.27, *t*=5.06, *p*<0.001) (see **Figure 2**).

As could be seen from the results of **Table 3**, the direct effect of cyber-ostracism on aggression was significant (direct effect size c ‘was 0.27, *SE*=0.05, 95% confidence interval is [0.16, 0.37]), 95% confidence interval did not contain 0. The mediating effect of the total score of basic psychological needs satisfaction was significant (the mediating effect size a*b was 0.17, *SE*=0.03, 95% confidence interval was [0.11, 0.24]), and the 95% confidence interval did not contain 0, indicating that the basic psychological needs satisfaction played an incomplete mediating role in the relationship between cyber-ostracism and aggression. The total effect size was c=a*b+c ‘=0.44, and it could be concluded that the mediating effect ratio of basic psychological needs satisfaction to the total effect was a*b/c=38.6%. The intermediary model diagram as follows:

### 3.3 The moderating effects of self-integrity

Model 14 of the process plug-in in SPSS25.0 was selected to conduct a moderated mediation model analysis to test whether the latter half of the mediating role of basic psychological needs satisfaction in the relationship between cyber-ostracism and aggression was moderated by self-integrity. The results are shown in **Table 4**.

**Table 4:** Testing the moderated mediating effect of cyber-ostracism and aggression.

Model (*N*=377)		Fit index			Significance	
Outcome variable	Predictor variable	*R*	*R^2^*	*F*	*β*	*t*
Basic psychological needs satisfaction		0.58	0.33	46.77^***^		
	Cyber-ostracism				−0.54	−12.82^***^
	Gender				0.14	1.611
	Age				−0.01	−0.28
	Frequency of SNS use				0.14	3.43^***^
Aggression		0.56	0.32	24.40^***^		
	Cyber-ostracism				0.32	5.90^***^
	Basic psychological needs satisfaction				−0.21	−3.30^**^
	Self-integrity				−0.14	−2.74^**^
	Basic psychological needs satisfaction * Self-integrity				0.14	3.34^***^
	Gender				0.11	1.28
	Age				0.01	0.21
	Frequency of SNS use				−0.03	−0.79

**Figure 3:**
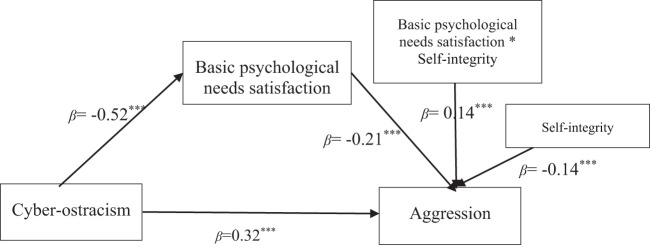
The moderated mediating effect of cyber-ostracism and aggression. ***p <.001.

As can be seen from the results in **Table 4**, cyber-ostracism significantly negatively predicted basic psychological needs satisfaction (*β*=−0.54, *t*=−12.82, *p*<0.001), the basic psychological needs satisfaction to meet a significant negative predictor of aggression(*β*=−0.21, *t*=−3.30, *p*<0.01), cyber-ostracism significantly positively predicted aggression(*β*=0.32, *t*=5.90, p<0.001); Self-integrity significantly negatively predicted aggression (*β*=−0.14, *t*=−2.74, *p*<0.01); The interaction terms of basic psychological needs satisfaction and self-integrity significantly positively predicted aggression (*β*=0.14, *t*=3.34, *p*<0.001) (see **Figure 3**). The mediated model diagram with adjustment as follows:

One standard deviation higher than the mean score of self-integrity (M+1SD) was taken as the group with high self-integrity, and one standard deviation lower than the mean score of self-integrity was taken as the group with low self-integrity (M-1SD). In different groups of self-integrity, the mediating effects of basic psychological needs satisfaction between cyber-ostracism and aggression were shown in **Table 5**.

**Table 5:** The mediating effect of different self-integrity groups.

Self-integrity	Relative value	*SE*	Bootstrap LLCI	Bootstrap ULCI
Low self-integrity	0.22	0.05	0.12	0.30
High self-integrity	0.04	0.05	−0.07	0.13

**Figure 4:**
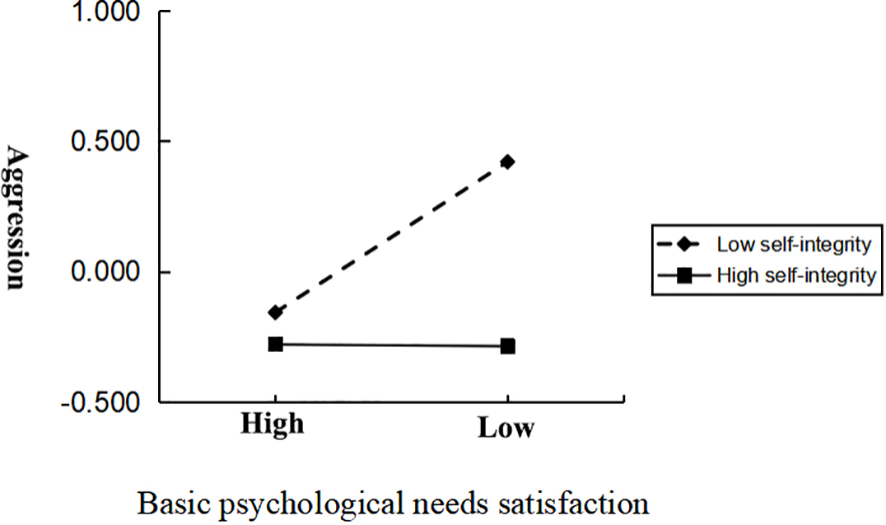
The association between basic psychological needs satisfaction and aggression for different level of self-integrity.

A simple slope analysis method was further used to explore the moderating effect of self-integrity on basic psychological needs satisfaction and aggression, and the results were shown in **Figure 4**. As could be seen from the slope chart, among college students with low self-integrity, aggression increased with the decrease of their basic psychological needs satisfaction, while among college students with high self-integrity, the increase trend of aggression slowed down with the decrease of their basic psychological needs satisfaction. Moreover, the results showed that when self-integrity was low (M-1SD), the basic psychological need to meet the significant negative prediction of aggression (*β*=−0.40, *t*=−4.89, *p*<0.001), when self-integrity was high (M+1SD), basic psychological needs satisfaction was not significant in predicting aggression(*β*=−0.07, *t*=−0.91, *p*>0.05).

## 4 Discussion

As one of the manifestations of maladjustment, aggressive behavior had been a research hotspot in the field of mental health. Studies of primary and secondary school students had found an adverse relationship between aggressive behavior and an individual’s social and emotional health (39, 40), therefore, it was very important to pay attention to the generation mechanism and protective factors of aggressive behavior. The Internet was the main platform for college students to socialize. Due to the anonymity of the Internet and the lack of social clues, the occurrence of network rejection was more and more common. The negative experience generated by cyber-ostracism was likely to prompt individuals to act aggressively (41). Based on self-determination theory and self-affirmation theory, this study explored the internal mechanism and protective factors of cyber-ostracism and aggressive behavior of college students. The results show that the basic psychological needs satisfaction played a partial mediating role between cyber-ostracism and aggression, and self-integrity moderates the latter half of the mediating role between basic psychological needs satisfaction and aggression. These results provide ideas for the study and intervention of college students’ aggressive behavior in the Internet age.

### 4.1 Cyber-ostracism and aggressive behavior

First, the results showed that college students’ experiences of cyber-ostracism could positively predict aggression. The conclusions of the relevant empirical studies are also consistent with our results. Social ostracism could increase individual aggression (42–44), and later experimental studies found that social ostracism in cyberspace would also lead to an increase in individual aggressive behavior (9, 45). For example, DeBono and Muraven’s (46) experimental study found that feelings of disrespect caused by social ostracism could predict individual aggression; Chen et al. (47) found that ostracism could predict aggression through the mediated model, and fatalistic belief played a moderating role. Nathan DeWall et al. (48) also found that rejection would lead to attacks on innocent people, but acceptance from others could ease the pain of rejection.

According to the temporal need-threat model of ostracism, individuals go through three stages after experiencing ostracism. In the second stage of reflection, individuals attempt to understand the ostracism event and recover from the social harm. One of the behavioral patterns that ostracized individuals commonly adopt is antisocial behavior, often manifested as aggression (41). Specifically, the experience of pain after ostracism (8) drives them to engage in aggression, which improves their mood and makes them feel better (49). The feeling of anger also plays a mediating role in their aggression following the experience of ostracism (50). In addition, aggression can also serve as a retaliatory response to alleviate their emotions (51).

The subjects of this study are college students. With the popularization of the Internet, online socialization among college students has become a widespread phenomenon. Due to the convenience and breadth of online social interaction, as well as its characteristics such as anonymity, lack of social cues, and fast spread of media, online ostracism may cause more severe harm to individuals compared to face-to-face ostracism (52). Ostracism experiences are often closely related to individuals’ aggression tendencies (43). One of the critical stages for inhibiting aggression is adolescence to early adulthood (53), which is the developmental stage of college students. Therefore, focusing on the impact of online ostracism on aggression and its mechanisms among college students can guide the design of interventions for ostracism experiences and have significant implications for mitigating aggression.

### 4.2 The mediating role of basic psychological needs satisfaction

Second, this study also found that the satisfaction of basic psychological needs partially mediates the relationship between cyber-ostracism and aggression. In other words, cyber-ostracism can indirectly predict aggression among college students by influencing the extent to which their basic psychological needs are satisfied. The self-determination theory proposes that individuals have three basic needs: the need for competence, the need for relatedness, and the need for autonomy. These needs are inherent growth tendencies and intrinsic psychological needs that form the foundation for self-motivation and personality integration (17). The satisfaction of individuals’ basic psychological needs is like psychological nutrients that serve as the foundation for their well-being and psychological growth, as well as important predictors of their behavioral responses (17). However, frustration in the satisfaction of basic psychological needs can lead to increased defensive reactions and pathological psychology (18). Therefore, when cyber-ostracism results in a decrease in the satisfaction of an individual’s basic psychological needs, defensive reactions may arise, leading to an increase in aggressive behavior.

This is consistent with previous research results. Cyber-ostracism is a common life situation that has negative effects on individuals’ physical and mental health, such as depression, anxiety, and reduced well-being, which can hinder the satisfaction of basic psychological needs (54). Therefore, due to social ostracism on the Internet, individuals are more likely to experience frustration in the satisfaction of their basic psychological needs, hindering their psychological growth and reducing their sense of well-being. Wang et al. (11) confirmed the negative predictive role of cyber-ostracism on the satisfaction of basic psychological needs. Through the experimental paradigm of online ostracism, they found that the satisfaction of basic psychological needs mediates the relationship between cyber-ostracism and sense of well-being. Cetin (55) conducted research on sports students and found that the higher the satisfaction of their basic psychological needs, the lower their aggression. The research by Kuzucu & Şimşek (56) also indicated a negative correlation between the satisfaction of basic psychological needs and aggression.

### 4.3 The moderating role of self-integrity

Third, the results of this study found that in college students with low self-integrity, the mediating effect between the satisfaction of basic psychological needs and self-integrity and aggression is significant. When self-integrity is low, the satisfaction of basic psychological needs significantly negatively predicts aggression; when self-integrity is high, the predictive effect of the satisfaction of basic psychological needs on aggression is not significant. According to the theory of self-affirmation, self-integrity refers to individuals generally feeling that they are good and socially adaptable (30). Threatening situations can damage an individual’s self-integrity, and individuals with high self-integrity can maintain their self-integrity by affirming self-worth unrelated to the threat domain, avoiding defensive reactions (31).

When individuals face threatening situations and their basic psychological needs are obstructed, if they have sufficiently self-integrity, they can maintain a positive self-evaluation by affirming other self-values unrelated to the threat domain, thereby reducing or even avoiding antisocial behavioral responses, and can learn about their own deficiencies in constructive ways to cope with threatening situations. Therefore, when individuals have high self-integrity, the negative predictive effect of the satisfaction of basic psychological needs on aggression is not significant.

In fact, the obstruction of basic psychological needs hinders self-integrity (17), and the results of this study also found a significant positive correlation between self-integrity and the satisfaction of basic psychological needs. However, in those individuals with high self-integrity, they may be better able to engage in “self-defense” (31) and offset the negative consequences brought by the obstruction of basic psychological needs.

Furthermore, self-affirmation maintains self-integrity by affirming self-worth unrelated to the threat domain. Among the three behavioral responses to social exclusion, only self-affirmation has the best social adaptability, which allows individuals to benefit from threatening information without undermining their own self-integrity (31). Individuals with high self-integrity may be more likely to adopt self-affirmation strategies to cope with the obstruction of basic psychological needs caused by social exclusion in the online society, thereby buffering the positive impact of the obstruction of basic psychological needs on aggression.

### 4.4 Implications and limitations

The theoretical significance of this study was that it provided a new research perspective for alleviating the negative impact of cyber-ostracism of college students in the Internet age. It also extended the study of the influence of cyber-ostracism on aggression, especially reveals the mechanism of the two variables. In practice, when the experience of cyber-ostracism increased, the degree of basic psychological needs satisfaction decreases and the tendency of aggressive behavior increased. At this time, self-integrity played a buffering role, reducing or even eliminating the tendency of college students to increase aggressive behavior due to the obstruction of basic psychological needs satisfaction. Self-integrity did not directly reduce the psychological threat felt by individuals in threatening situations, but alleviated or even eliminated the negative effects brought by these psychological threats (16). Thus, we could see the importance of self-integrity in regulating individual behavior under threat situations. In everyday school education, it is possible to incorporate psychological courses and group counseling sessions that are related to self-integrity. When individuals face cyber-ostracism, psychologists or counselors can guide and assist them in enhancing their self-integrity. These findings provided implications for interventions to reduce aggressive behavior and buffer negative emotions caused by cyber-ostracism.

This study also has certain limitations that need to be further addressed and expanded upon in future research. Firstly, this study primarily focused on college students as the research subjects. However, according to the 51st China Internet Development Statistics Report, the number of young and elderly the Internet users in China is increasing year by year (7). Research has shown that levels of aggression in individuals during adolescence follow a curvilinear trend, reaching their peak around the age of 14-15 (57). The development of self-integrity also occurs gradually during adolescence. For adolescents, it is crucial to control factors that may trigger aggressive behavior, promote the development of self-integrity, and alleviate the impacts of online ostracism. Furthermore, due to aging, older adults perceive time as limited, resulting in a greater focus on current important intimate relationships (58). This perception difference in interpersonal relationships further leads to different reactions to cyber-ostracism, warranting further exploration. Therefore, the role of cyber-ostracism and self-integrity in young and older population groups is also worth investigating in future research.Secondly, this study adopted a cross-sectional design, which cannot establish causal relationships. Future research can employ experimental designs to manipulate cyber-ostracism and explore the causal relationships between these variables, conducting intervention studies if possible. Thirdly, ostracism in real-life contexts is multifaceted and can be differentiated into neglect and exclusion. Are there different types of cyber-ostracism? This study only focused on the broad phenomenon of cyber-ostracism. Future research can consider exploring the effects of different types of cyber-ostracism on individuals’ psychological and behavioral outcomes, or adopt specific paradigms to study the effects of cyber-ostracism in specific domains, such as social media ostracism (28). It is essential to enrich the empirical research in the field of cyber-ostracism.

## Data availability statement

The raw data supporting the conclusions of this article will be made available by the authors, without undue reservation.

## Ethics statement

The studies involving humans were approved by The ethics committee of Central China Normal University. The studies were conducted in accordance with the local legislation and institutional requirements. The participants provided their written informed consent to participate in this study.

## Author contributions

JX: Writing – review & editing, Writing – original draft, Validation, Resources, Project administration, Methodology, Investigation, Funding acquisition, Formal analysis, Conceptualization. FK: Writing – review & editing, Visualization, Software, Resources, Methodology, Investigation, Data curation.
